# New Glomeromycotan Taxa, *Dominikia glomerocarpica* sp. nov. and *Epigeocarpum crypticum* gen. nov. et sp. nov. From Brazil, and *Silvaspora* gen. nov. From New Caledonia

**DOI:** 10.3389/fmicb.2021.655910

**Published:** 2021-04-23

**Authors:** Janusz Błaszkowski, Khadija Jobim, Piotr Niezgoda, Edward Meller, Ryszard Malinowski, Paweł Milczarski, Szymon Zubek, Franco Magurno, Leonardo Casieri, Wojciech Bierza, Tomasz Błaszkowski, Thomas Crossay, Bruno Tomio Goto

**Affiliations:** ^1^Laboratory of Plant Protection, Department of Shaping of Environment, West Pomeranian University of Technology in Szczecin, Szczecin, Poland; ^2^Departamento de Botânica e Zoologia, Universidade Federal do Rio Grande do Norte, Natal, Brazil; ^3^Laboratory of Soil Science and Environmental Chemistry, Department of Shaping of Environment, West Pomeranian University of Technology in Szczecin, Szczecin, Poland; ^4^Department of Genetics, Plant Breeding and Biotechnology, West Pomeranian University of Technology in Szczecin, Szczecin, Poland; ^5^Faculty of Biology, Institute of Botany, Jagiellonian University, Kraków, Poland; ^6^Institute of Biology, Biotechnology and Environmental Protection, Faculty of Natural Sciences, University of Silesia in Katowice, Katowice, Poland; ^7^Mycorrhizal Applications LLC at Bio-Research & Development Growth Park, St. Louis, MO, United States; ^8^Department of General and Oncological Surgery, Pomeranian Medical University in Szczecin, Szczecin, Poland; ^9^Institut des Sciences Exactes et Appliquées (EA 7484), Université de la Nouvelle Calédonie, Nouméa, New Caledonia

**Keywords:** arbuscular mycorrhizal fungi, cryptic species, morphology, molecular phylogeny, 18S-ITS-28S nuc rDNA, *rpb1*

## Abstract

Examination of fungal specimens collected in the Atlantic rain forest ecosystems of Northeast Brazil revealed many potentially new epigeous and semihypogeous glomerocarp-producing species of the phylum Glomeromycota. Among them were two fungi that formed unorganized epigeous glomerocarps with glomoid spores of almost identical morphology. The sole structure that distinguished the two fungi was the laminate layer 2 of their three-layered spore wall, which in spores of the second fungus crushed in PVLG-based mountants contracted and, consequently, transferred into a crown-like structure. Surprisingly, phylogenetic analyses of sequences of the 18S-ITS-28S nuc rDNA and the *rpb1* gene indicated that these glomerocarps represent two strongly divergent undescribed species in the family Glomeraceae. The analyses placed the first in the genus *Dominikia*, and the second in a sister clade to the monospecific generic clade *Kamienskia* with *Kamienskia bistrata*. The first species was described here as *Dominikia glomerocarpica* sp. nov. Because *D. glomerocarpica* is the first glomerocarp-forming species in *Dominikia*, the generic description of this genus was emended. The very large phylogenetic distance and the fundamental morphological differences between the second species and *K. bistrata* suggested us to introduce a new genus, here named as *Epigeocarpum* gen. nov., and name the new species *Epigeocarpum crypticum* sp. nov. In addition, our analyses also focused on an arbuscular mycorrhizal fungus originally described as *Rhizophagus neocaledonicus*, later transferred to the genus *Rhizoglomus*. The analyses indicated that this species does not belong to any of these two genera but represents a new clade at the rank of genus in the Glomeraceae, here described as *Silvaspora* gen. nov.

## Introduction

In the phylum Glomeromycota, the largest group is represented by species producing glomoid spores, which arise blastically at tips of cylindrical or funnel-shaped sporogenous hyphae, as spores of *Glomus macrocarpum* (see Supplementary Table 1 for species authors), the type species of *Glomus* and the Glomeromycota (Clements and Shear, 1931; Schüßler and Walker, 2010; Oehl et al., 2011). Of the 330 known species of the Glomeromycota, approximately 60 were originally described to form glomoid spores in epigeous or hypogeous unorganized glomerocarps, i.e., fruit bodies with randomly distributed spores inside them (Gerdemann and Trappe, 1974; Morton, 1988; Jobim et al., 2019). Importantly, of the glomerocarpic species, only seven (*Diversispora epigaea*, *Funneliformis mosseae*, *Glomus arborense*, *G. macrocarpum*, *Glomus pallidum*, *Glomus tenerum*, and *Glomus warcupii*) were managed to grow in culture, and only eight (*D. epigaea*, *Diversispora sporocarpia*, *G. macrocarpum*, *Redeckera megalocarpa*, *Redeckera fulva*, *Redeckera pulvinata*, *Sclerocarpum amazonicum*, and *Sclerocystis sinuosa*) were provided with molecular data (Jobim et al., 2019), which essentially makes this group of fungi ascribed to the Glomeromycota difficult to characterize and classify.

Among many unorganized glomerocarps, previously found from our studies in the Brazilian Northeast, there were two whose glomoid spores first seemed to be identical in morphology to each another but differed from glomoid spores of described species of the Glomeromycota. Further studies of the phenotypic features of the spores revealed very small differences and, consequently, suggested that the glomerocarps represent two semicryptic undescribed species. BLAST searchers using initial molecular data about these fungi confirmed this hypothesis and showed that these species are probably related to the genera *Dominikia* and *Kamienskia* in the family Glomeraceae.

“Crypticity” in fungal taxonomy is an aspect known for several phyla (Matute and Sepúlveda, 2019). Also, molecular analyses of some morphological species of the Glomeromycota suggest that they contain cryptic phylogenetic species, in which differences in morphology do not occur, are very small and inconclusive, or are invisible using traditional research methods and tools. Examples of such species are *Rhizoglomus irregulare* and *Septoglomus constrictum* (Stockinger et al., 2014; Błaszkowski, pers. observ.).

The disclosure of cryptic species strongly depends on the molecular markers used in phylogenetic identification. Currently, of the many markers that have been used in phylogenetic studies of the Glomeromycota (Błaszkowski et al., 2018a, b, 2019a, b; Jobim et al., 2019; Magurno et al., 2019), those comprising the partial small subunit (18S), internal transcribed spacer (ITS1-5.8S-ITS2 = ITS), and partial large subunit (28S) nuc rDNA segment (= 18S-ITS-28S), as well as the largest subunit of RNA polymerase II (*rpb1*) gene, have the highest resolution power and characterize the highest proportion of described species within the Glomeromycota (Krüger et al., 2012; Stockinger et al., 2014; Al-Yahya’ei et al., 2017). The first marker allowed to separate, for example, *Rhizoglomus dunense* from *Rhizoglomus clarum*, whose morphologies are almost identical, but sequence divergence is very high (13.3%; Al-Yahya’ei et al., 2017). The sequence resolution of the *rpb1* gene is higher and indicating that *R. irregulare* likely contains at least one cryptic species (Stockinger et al., 2014) and strongly confirming the uniqueness of *R. dunense* with a dissimilarity of 8.1% from *R. clarum*. According to literature data and our analyses, 18S-ITS-28S and *rpb1* sequences show interspecies differences when they differ by ≥3% and approximately 1%, respectively (Krüger et al., 2012; Stockinger et al., 2014; Błaszkowski et al., 2019b; Corazon-Guivin et al., 2019b). The large proportion of species provided with molecular data contained in the two markers allows both to compare a relatively large number of species and to better define the phylogenetic divergences between species belonging to different higher taxonomic ranks. Moreover, it is widely recognized that the concatenation of sequences of the ribosomal RNA-coding genes with sequences of protein-coding genes significantly increases phylogenetic resolving power for revealing relationships between analyzed taxa (Miadlikowska et al., 2014).

The genera *Dominikia* and *Kamienskia* were erected in the phylum Glomeromycota mainly due to phylogenetic analyses of 18S-ITS-28S sequences of six species originally described in the genus *Glomus* and the newly described *Dominikia disticha* (Błaszkowski et al., 2015a). The type species of these new genera became *Dominikia minuta* and *Kamienskia bistrata*. The main characters shared by the two fungi were hyaline or pale-colored, small spores (10- to 65-μm diameter when globose) formed in hypogeous clusters, which were produced in pot trap cultures, but were not found in the field-collected rhizosphere samples used to inoculate the cultures. Therefore, it was suggested that species of *Dominikia* and *Kamienskia* sporulate rarely or seasonally and were not found in the field soils because their delicate spores quickly break down before soil sampling (Błaszkowski et al., 2015a).

Later, new species of *Dominikia* and *Kamienskia* were described, whose spores were either (i) similar in color and size (*Dominikia lithuanica*, *Dominikia litorea*, *Kamienskia divaricata*), (ii) darker-colored (*Dominikia aurea*, *Dominikia bernensis*, *Dominikia compressa*, *Dominikia duoreactiva*, *Dominikia emiratia*), (iii) larger (*D. compressa*, *D. duoreactiva*, *D. emiratia*), and (iv) formed mainly singly (*Dominikia difficilevidera*) (Oehl et al., 2014, 2015; Błaszkowski et al., 2015b, 2016, 2018c; Al-Yahya’ei et al., 2017) compared to the spores of the species listed above.

Recently, *Kamienskia perpusilla* and *K. divaricata* were transferred to a newly erected genus, *Microkamienskia* with a newly described species, *Microkamienskia peruviana* (Corazon-Guivin et al., 2019a), and *D. litorea* and *D. emiratia* became members of the new genera *Microdominikia* and *Orientoglomus*, respectively (Corazon-Guivin et al., 2019b). In addition, Corazon-Guivin et al. (2019b) described a new, monospecific genus, *Nanoglomus*, with *Nanoglomus plukenetiae*, sp. nov., with very similar morphology to *K. perpusilla*, but phylogenetically closely related to species of clades previously treated as *Dominikia*. All the taxa were erected mainly based on phylogenetic analyses of 18S-ITS-28S sequences and divergences of the sequences from other most closely related species, although Corazon-Guivin et al. (2019b) also showed morphological differences in the spore wall and subtending hypha between these taxa.

All described species of *Dominikia* and *Kamienskia* and their closest molecular relatives possess morphological characters distinguishing them from each other. However, none of these species has a morphological synapomorphy that would clearly demonstrate a generic separateness.

Recently, a new AMF, namely, *Rhizophagus neocaledonicus*, was described, which in the field lived in symbiosis with metallophytes in ultramafic soil in New Caledonia (Crossay et al., 2018). It was soon reassigned to the genus *Rhizoglomus*, as *Rhizoglomus neocaledonicum* (Turrini et al., 2018), because the generic name *Rhizophagus* should not be assigned to any species in the Glomeromycota (Sieverding et al., 2014). The assignment of this species into the genus *Rhizoglomus* (treated as *Rhizophagus*) was made based on a maximum likelihood phylogenetic analysis of 18S-ITS-28S sequences, even though the *Rhizoglomus* clade with the basally positioned *R. neocaledonicum* obtained only 49% bootstrap support. Moreover, the genetic divergence of *R. neocaledonicum* from the six *Rhizoglomus* species considered in the analysis was in the order of values separating clades of the Glomeromycota at the rank of genus. The sister species to the *Rhizoglomus* clade was a member of the genus *Sclerocystis*, *S. sinuosa* (Crossay et al., 2018). Molecular phylogenetic analyses performed by Turrini et al. (2018) confirmed the basal position and strong genetic divergence of *R. neocaledonicum* in the *Rhizoglomus* clade. However, they did not include *S. sinuosa* in their analyses. Further phylogenetic analyses of 18S-ITS-28S sequences of all *Rhizoglomus* species with available DNA barcodes and representatives of the other genera of the Glomeraceae accommodated *R. neocaledonicum* between the recently described monospecific genus *Halonatospora*, represented by *Halonatospora pansihalos*, and *S. sinuosa*, of which the latter was located basally to the other species of *Rhizoglomus* (Błaszkowski et al., 2018b, 2019b). Thus, the information presented above suggested that the New Caledonian fungus does not belong to *Rhizoglomus* but represents a new clade at the rank of genus in the Glomeraceae.

Therefore, the aims of this study were (i) to document that the two Brazilian fungi are new species of the Glomeromycota, (ii) to determine the molecular phylogenetic position of these fungi among their generic relatives of known phylogenies, and (iii) to describe in detail the morphology of these fungi. Moreover, we tested the hypothesis that *R. neocaledonicum* is a member of an undescribed genus in the Glomeraceae using already available and newly collected morphological and molecular data about this species.

## Materials and Methods

### Origin of Study Material

The glomerocarps of the two fungi were sampled by K. Jobim at Parque das Trilhas, belonging to the Serra de Baturité Environmental Protection Area (Baturité EPA; 4°16′36.8′′ S, 38°56′20.4′′ W; 865 m above sea level) in the Guaramiranga municipality. The area covers 32,690 hectares located in the Northeast portion of the State of Ceará, 90 km from Fortaleza (Governo do Ceará, 2018) and is part of the Atlantic Forest. It houses a rich biodiversity with a high degree of endemism and includes a high percentage of preserved remnants of the Atlantic Forest that constitute a refuge for species originating from humid habitats, such as the Atlantic and Amazon forests (Figueiredo and Barbosa, 1990; Girão, 2006). In this region, annual mean temperatures are 19–22°C, and the average annual sum of rainfalls is 1,500 mm (Governo do Ceará, 2018). The high rainfalls, the altitude range between 100 and 1,000 m above sea level, and the exposure to humid air masses render the Baturité EPA one of the most humid areas in the State of Ceará (Governo do Ceará, 2018). The glomerocarps of these species, tentatively named “Species 1” and “Species 2,” were found in July 2018 and April 2018, respectively.

### Collection of Glomerocarps, Establishment, and Growth of Single-Species Cultures

The glomerocarps of the two fungi were found employing the methodology used by Jobim et al. (2019) for collecting glomerocarpic species. Single-species pot cultures for each of the collected fungi were established by inoculating sterile growth substrate with fragments of the glomerocarps, of which each fragment contained at least 100 spores. The plant host used in the pot cultures was *Plantago lanceolata* L. The composition of the growth substrate, the methods of culture establishment, inoculation, and growing conditions were as those described by Błaszkowski et al. (2012).

### Extraction of Spores and Staining of Mycorrhizal Structures

The spore conglomerations used for the establishment of single-species cultures were extracted from the glomerocarps by means of a preparation needle under a dissecting microscope. Attempts at extracting spores from the single-species cultures of the two fungi were performed by a method characterized by Błaszkowski et al. (2015b). Roots of *P. lanceolata* from the single-species cultures were stained following the protocol of Błaszkowski (2012).

### Microscopy and Nomenclature

Morphological features of spores and phenotypic and histochemical characters of spore wall layers of the two fungi were characterized based on at least 100 spores of each species mounted in water, lactic acid, polyvinyl alcohol/lactic acid/glycerol (PVLG, Omar et al., 1979), and a mixture of PVLG and Melzer’s reagent (1:1, vol/vol). The preparation of spores for study and photography were as those described previously (Błaszkowski, 2012; Błaszkowski et al., 2012). Types of spore wall layers are those defined by Walker (1983) and Błaszkowski (2012). Color names were from Kornerup and Wanscher (1983). Nomenclature of fungi and the authors of fungal names are from the Index Fungorum website, http://www.indexfungorum.org/AuthorsOfFungalNames.htm; see Supplementary Table 1. The terms “glomerospores” and “glomerocarps” were used for spores and fruit bodies produced by AMF, as proposed by Goto and Maia (2006) and Jobim et al. (2019).

Voucher specimens of the proposed new species [spores permanently mounted in PVLG and a mixture of PVLG and Melzer’s reagent (1:1, vol/vol) on slides] were deposited at UFRN hebarium (Brazil; holotypes on slides and dry glomerocarps in vials) and in the Laboratory of Plant Protection, Department of Shaping of Environment (LPPDSE; isotypes on slides and dry glomerocarps in vials), West Pomeranian University of Technology in Szczecin, Poland.

### DNA Extraction, Polymerase Chain Reaction, Cloning, and DNA Sequencing

Genomic DNA of the two potentially new species was extracted separately from fragments of glomerocarps containing approximately 20 spores. Details on spores preparation prior to polymerase chain reaction (PCR), PCR conditions, and primers used to obtain 18S-ITS-28S amplicons were as described in Krüger et al. (2009), Błaszkowski et al. (2014), and Symanczik et al. (2014), respectively.

The *rpb1* sequences of *Rhizoglomus dalpeae* were obtained by amplification with primers designed by Stockinger et al. (2014) following the recommended conditions. We used the same DNA, from which 18S-ITS-28S sequences had been obtained (Błaszkowski et al., 2019b). The first PCR with DNA of this species was performed with the primers RPB1-DR160mix (a, b, c, d) and RPB1-HS2680GPr, whereas the second PCR with RPB1-HS189GPf and RPB1-DR1210r.

The *rpb1* sequences of “Species 1” and “Species 2” were amplified with a nested approach. The primer set consisted of two forward primers, RPB1-3F and RPB1-4F, on the third and fourth exon, respectively, and two reverse primers, RPB1-5R and RPB1-5RN, on the fifth exon of the gene (Table 1). The *rpb1* sequences of *R. neocaledonicum* and *Rhizoglomus silesianum* were amplified using the primer RPB1-4F1, designed on the fourth exon to match all the *rpb1* sequences available for the Glomeraceae (with a maximum tolerance of one mismatch), in combination with RPB1-5R. The three forward primers were used at a final concentration = 200 nM, whereas the concentration of reverse primers was higher (Table 1) to compensate the gap in the Tm predicted using Oligo/Analyzer^1^. DreamTaq DNA Polymerase (Thermo Fisher) was used for the amplifications in 20 μL final volume according to the manufacturer’s specifications, adding MgCl_2_ 3 mM and bovine serum albumin 0.5 μg μL^–1^ as final concentrations only in the PCR on raw DNA. The thermal cycling was as follows: 5 min initial denaturation (95°C), 40 cycles (30 cycles in the nested PCR) of 30-s denaturation (95°C), 30-s annealing, elongation at 72°C (Table 1), and 5-min (72°C) final elongation.

**TABLE 1:** Primers characteristics and PCR conditions used for the amplification of the *rpb1* sequences.

Primer	Nucleotide sequence (5′–3′)	Position on the sequence	In combination with	Expected amplicon length	Ta°C	t elong.
RPB1-3F	GTC TTC GTG CAG TTT GGG A	727–745	RPB1-5R [1 μM]	≈1,410 bp	55	1 min 30 s
RPB1-4F	CTA GGC CTG ATT GGA TGA T	1,204–1,222	RPB1-5RN [500 nM]	≈870 bp	54	1 min
RPB1-4F1	GCT CGT CCT GAT TGG ATG A	1,203–1,221	RPB1-5R [1 μM]	≈935 bp	55	1 min
RPB1-5R	ACG ATT TGT TTT GGT ACC AT	2,119–2,138				
RPB1-5RN	TTC ATC TCA TCA CCA TCA A	2,048–2,066				

*The position of primers is given based on sequence of Rhizoglomus intraradices HG316020.1. The final concentration of the reverse primers is provided for each combination, as well as the expected product size, the temperature of annealing (Ta), and the time of elongation (t elong).*

Cloning and sequencing of the PCR products were as those described by Błaszkowski et al. (2015a). The sequences were deposited in GenBank (MW507148–57, MW541060–67).

### Sequence Alignment and Phylogenetic Analyses

**FIGURE 1:**
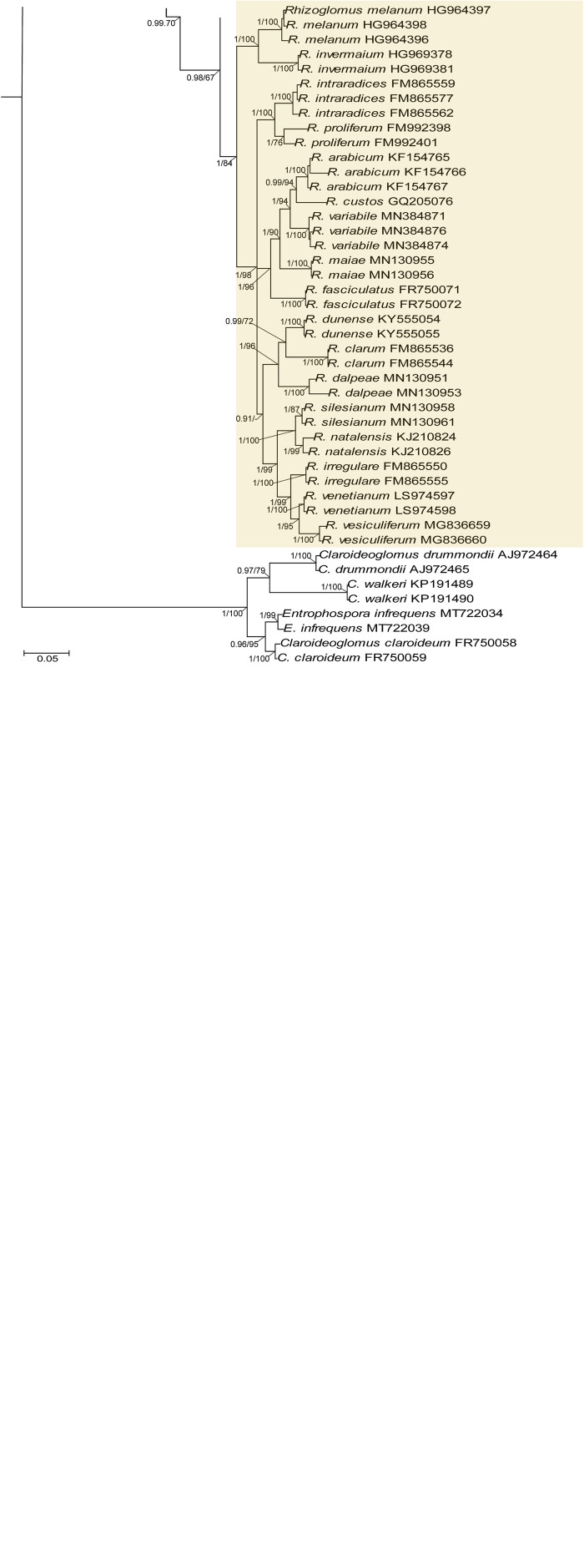
A 50% majority rule consensus phylogram inferred from a Bayesian inference analysis of 18S-ITS-28S nuc rDNA sequences of *Dominikia glomerocarpica* and *Epigeocarpum crypticum*, as well as 50 other species of AMF, including *Claroideoglomus claroideum*, *Claroideoglomus drummondii*, *Claroideoglomus walkerii*, and *Entrophospora infrequens* as outgroup. The Bayesian posterior probabilities ≥0.90 and ML bootstrap values ≥50% are shown near the branches, respectively. Bar indicates 0.05 expected change per site per branch.

**FIGURE 2:**
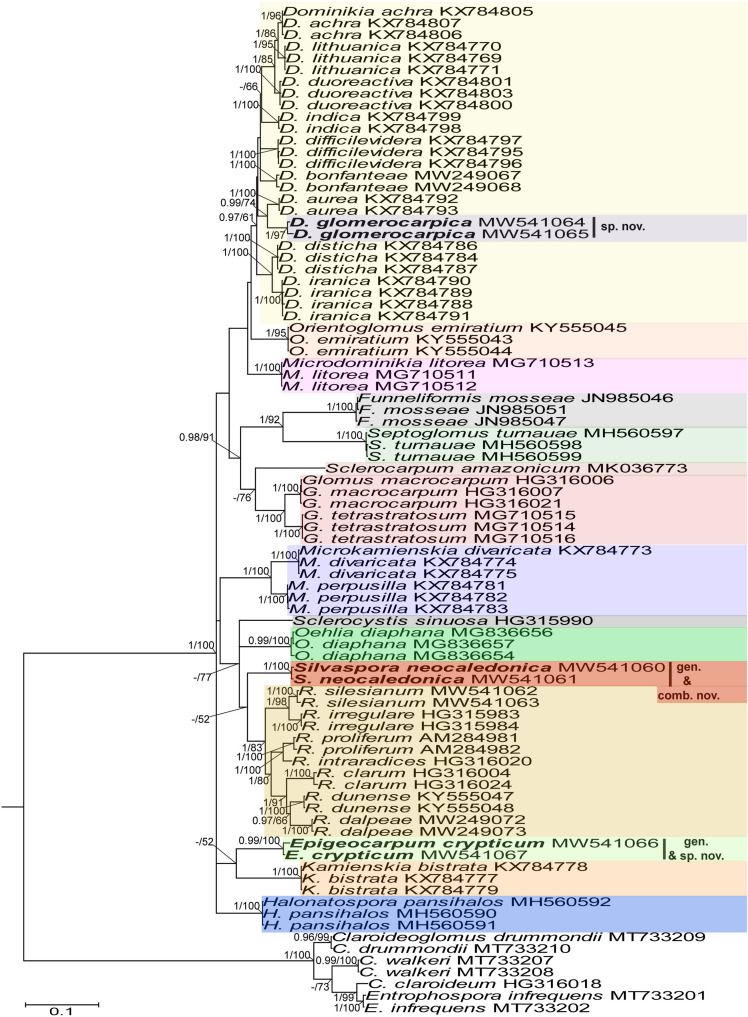
A 50% majority rule consensus phylogram inferred from a Bayesian inference analysis of *rpb1* sequences of *D. glomerocarpica* and *E. crypticum*, as well as 34 other species of AMF, including *C. claroideum*, *C. drummondii*, *C. walkerii*, and *E. infrequens* as outgroup. The Bayesian posterior probabilities ≥0.90 and ML bootstrap values ≥50% are shown near the branches, respectively. Bar indicates 0.1 expected change per site per branch.

**FIGURE 3:**
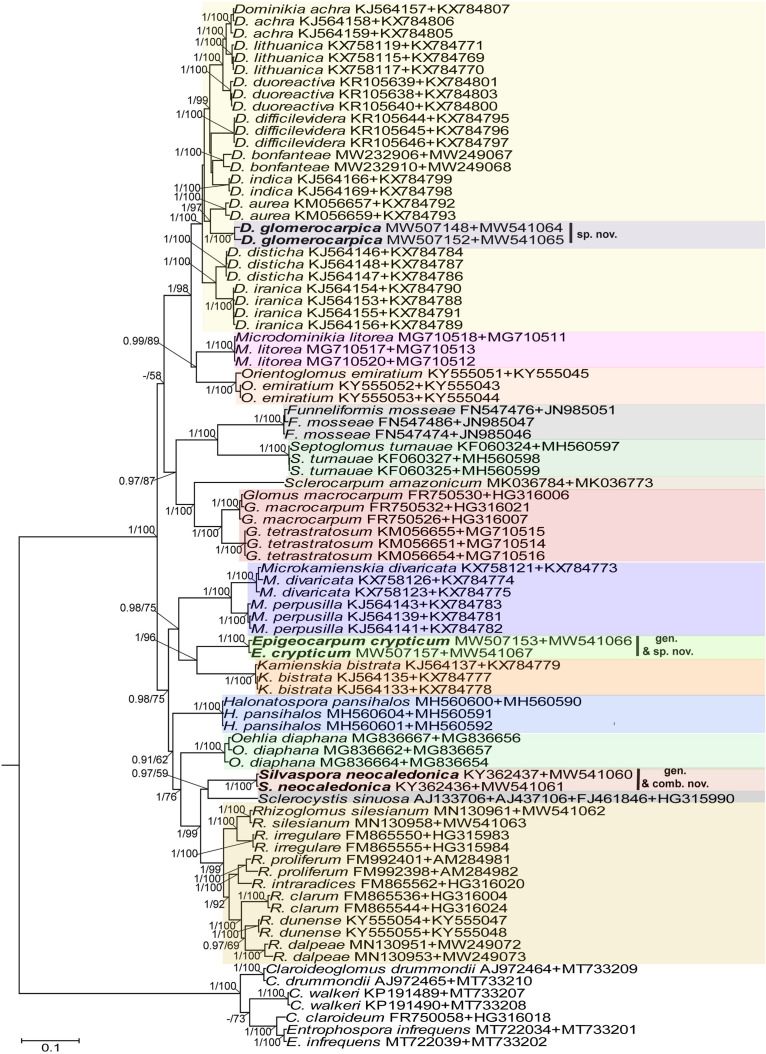
A 50% majority rule consensus phylogram inferred from a Bayesian inference analysis of 18S-ITS-28S + *rpb1* sequences of *D. glomerocarpica* and *E. crypticum*, as well as 34 other species of AMF, including *C. claroideum*, *C. drummondii*, *C. walkerii*, and *E. infrequens* as outgroup. The Bayesian posterior probabilities ≥0.90 and ML bootstrap values ≥50% are shown near the branches, respectively. Bar indicates 0.1 expected change per site per branch.

Comparative analyses of 18S-ITS-28S and *rpb1* sequences of the two fungi, using BLAST, confirmed our initial hypotheses that the fungi of our study are undescribed species of the Glomeromycota (see section “Introduction”). In particular, these analyses indicated that “Species 1” belongs to *Dominikia*, and the closest relative of “Species 2” is *K. bistrata*. Three sequence alignments were created in order (*i*) to determine the position of the new *Dominikia* sp. among its generic relatives, (*ii*) to assess the robustness of the relationship of the second new species with *K. bistrata*, and (*iii*) to test the hypothesis expressed previously (Błaszkowski et al., 2019b) and here that *R. neocaledonicum* should represent a new genus. The first alignment consisted of sequences of the 18S-ITS-28S nuc rDNA region or part thereof, which characterized all sequenced species of *Dominikia*, *Kamienskia*, and *Rhizoglomus*, as well as representative species of all other genera of the Glomeraceae *sensu* Schüßler and Walker (2010), Oehl et al. (2011), Błaszkowski et al. (2015a, 2018a, b), Corazon-Guivin et al. (2019a, b, c), Jobim et al. (2019), and Wijayawardene et al. (2020), except for the genus *Simiglomus* (a total of 15 genera; Figure 1). The alignment contained 139 sequences of 48 species of the Glomeraceae (including our two new species), of which the *Rhizoglomus natalense* KJ210824 and KJ210826 sequences represented the 28S locus only. To increase the phylogenetic signal from *S. sinuosa*, the 18S-ITS-28S sequence, not available in the GenBank database, was obtained by assembling the overlapping sequences AJ133706 (18S), AJ437106 (18S-ITS-28S), and FJ461846 (28S) generated from the same isolate MD126. The second alignment consisted of *rpb1* sequences from all species of the 18S-ITS-28S alignment that have been provided with *rpb1* sequences (Figure 2). This alignment included 75 sequences of 29 species in 14 genera of the Glomeraceae (including our two new species). The third alignment contained concatenated 18S-ITS-28S and *rpb1* sequences in the same number and species composition as in the *rpb1* alignment (Figure 3). In all three alignments, the outgroup was represented by seven sequences of four species of the family Entrophosporaceae. Data about the origin of the sequences used are presented in Supplementary Table 2.

Identity values of the 18S-ITS-28S and *rpb1* sequences of the two new species were calculated separately for each species using BioEdit (Hall, 1999). With the same program, we calculated the sequence divergence of these new species and *R. neocaledonicum* from sequences of their closest relatives (Figures 1, 2). All comparisons were performed on sequences of the same length.

Indels were coded as binary characters by means of FastGap 1.2 (Borchsenius, 2009), with the possibility to code missing data to be recognized by the phylogenetic inference programs. The coded binary character sets were added to the respective nucleotide alignments, as described in Błaszkowski et al. (2014). The 18S-ITS-28S, *rpb1*, and 18S-ITS-28S + *rpb1* alignments were aligned separately with MAFFT 7 using the auto option^2^.

In order to reconstruct the phylogenetic positions of the two new species among sequenced species of the Glomeraceae, as well as to confirm our hypothesis on the phylogeny of *R. neocaledonicum*, Bayesian inference (BI) and maximum likelihood (ML) phylogenetic analyses of the alignments were performed via CIPRES Science Gateway 3.1 (Miller et al., 2010). GTR + G was predicted as best substitution model for the DNA partitions in the BI analysis by using jModelTest2 (Darriba et al., 2012). For the indel partition, F81 model was chosen as suggested in the MrBayes manual. Four Markov chains were run over 1 million generations in MrBayes 3.2 (Ronquist et al., 2012), sampling every 1,000 generations, with a burn-in at 3,000 sampled trees. The ML phylogenetic tree inference, using a maximum likelihood/1,000 rapid bootstrapping run, was computed with RAxML 8.2.12 (Stamatakis, 2014) using the GTRGAMMA algorithm. To improve the accuracy of phylogenetic reconstruction (Lanfear et al., 2012; Nagy et al., 2012), in both BI and ML analyses, the 18S-ITS-28S alignment was divided into four partitions: 18S, ITS, 28S, and the binary (indel) character set. Two partitions (gene and indel set) were used for the *rpb1* alignment. The same partitioning scheme was used in the 18S-ITS-28S + *rpb1* alignment with concatenated sequences, resulting in six partitions totally. We assumed that clades were supported when BI posterior probability and ML bootstrap support values were ≥0.95 and ≥70%, respectively. The phylogenetic trees obtained in the analyses were visualized and rooted in Archaeopteryx.js^3^.

## Results

### General Data and Phylogeny

Data about the numbers of base pairs as well as variable and parsimony informative sites of the 18S-ITS-28S, *rpb1*, and 18S-ITS-28S + *rpb1* alignments are presented in Table 2. The identity values of the 18S-ITS-28S sequences of “Species 1” and “Species 2” (five sequences each) were 98.6 and 99.4%, respectively, and the identity values of the *rpb1* sequences of these species (two sequences each) were 99.1%. The identities of the three 18S-ITS-28S and two *rpb1* sequences of *Rhizoglomus neocaledonicum* were 99.5 and 99.7%, respectively.

**TABLE 2:** Characteristics of the sequence alignments analyzed.

Name of alignment	Number of sequences	Number of species	Number of base pairs	Number of variable sites	Number of parsimony informative sites
18S-ITS-28S	147	52	2,492	1,094	978
*rpb1*	86	36	2,841	1,390	1,195
18S-ITS-28S + *rpb1*	86	36	4,074	2,350	2,067

In the 18S-ITS-28S and 18S-ITS-28S + *rpb1* trees, the topologies of the family Glomeraceae clade, reconstructed from BI and ML analyses, were identical (Figures 1, 3). Additionally, for all trees (Figures 1–3), the branches of the outgroup Entrophosporaceae clade received full or strong BI and ML support. Instead, the topologies of the *rpb1* Glomeraceae clade slightly differed depending on the algorithm used in the analyses. The differences regarded only the positions of the monospecific genera *Microdominikia* and *Orientoglomus* (Figure 2). In the *rpb1* BI tree, *Microdominikia litorea* was a basal clade to the clades with *Orientoglomus emiratium* and *Dominikia* spp. Instead, in the *rpb1* ML tree, the relationships between *Orientoglomus*, *Microdominikia*, and *Dominikia* clades were not supported (Figure 2).

In all trees, 18S-ITS-28S, *rpb1*, 18S-ITS-28S + *rpb1*, “Species 1” (=*Dominikia glomerocarpica*) clustered in a sister clade to the clade with *D. aurea*, and “Species 2” (= *Epigeocarpum crypticum*) populated a sister clade to the clade with *K. bistrata* (Figures 1–3). Both BI and ML analyses of all three alignments supported the tie between “Species 1” and *D. aurea* (BI = 0.99–1.0; ML = 74–97%; Figures 1–3). Instead, the node connecting “Species 2” with *K. bistrata* obtained moderate or strong supports in BI and ML analyses of the 18S-ITS-28S (0.99/88) and 18S-ITS-28S + *rpb1* (1/96) alignments (Figures 1, 3), but neither BI nor ML analysis of the *rpb1* alignment supported (0.68/52) the tie between these species (Figure 2).

In the 18S-ITS-28S and 18S-ITS-28S + *rpb1* trees, the clades with “Species 1” and “Species 2” obtained full supports in both analyses, and in the *rpb1* tree, the supports for both species were full or high (BI = 1.0, ML = 97% for “Species 1” and BI = 0.99, ML = 100% for “Species 2”; Figures 1, 3).

The *R. neocaledonicum* (=*Silvaspora neocaledonica*) clade in the 18S-ITS-28S, *rpb1*, and 18S-ITS-28S + *rpb1* trees obtained full supports in both BI and ML analyses (Figures 1–3). In the 18S-ITS-28S and 18S-ITS-28S + *rpb1* trees, *R. neocaledonicum* was placed in a sister clade to *S. sinuosa* (Figures 1, 3). The node linking the two species was moderately supported in both BI and ML analyses of the 18S-ITS-28S alignment (BI = 0.97, ML = 80%), whereas only the BI analysis of the 18S-ITS-28S + *rpb1* alignment moderately (BI = 0.97) supported this association (Figures 1, 3). The closest neighbors of the *R. neocaledonicum–S. sinuosa* clade were the generic clades *Halonatospora* and *Rhizoglomus*, respectively. In the *rpb1* tree, *R. neocaledonicum* sequences clustered basally with weak support (BI = 0.77, ML = 52) to the *Rhizoglomus* spp. clade (Figure 2).

The molecular distances between “Species 1” and *D. aurea*, calculated based on comparisons of their 18S-ITS-28S and *rpb1* sequences, were 8.5–10.2% and 4.3–4.8%, respectively. The divergences of the 18S-ITS-28S and *rpb1* sequences of “Species 2” and *K. bistrata* were 14.5–15.6% and 11.0–11.6%, respectively. The 18S-ITS-28S and *rpb1* sequences of *R. neocaledonicum* and *S. sinuosa* differed by 22.7–22.9% and 11.4%, respectively. The 18S-ITS-28S and *rpb1* sequences of *R. neocaledonicum* and *Rhizoglomus* spp. differed by 12.5–18.5% and 10.8–13.0%, respectively.

Considering the results of the phylogenetic analyses and comparisons of sequences of the two species found in northeast Brazil and the AMF originally described as *Rhizophagus neocaledonicus* discussed above, as well as the morphological distinctiveness of these species from their closest phylogenetically relatives, below we characterize the two species from Brazil as *D. glomerocarpica* sp. nov. and *E. crypticum* gen. nov. et sp. nov. In addition, we emend the morphological description of and transfer *Rhizophagus neocaledonicum* to a new genus, *Silvaspora* gen. nov., with *S. neocaledonica* comb. nov. Because *D. glomerocarpica* is the first glomerocarp-forming species in *Dominikia*, the definition of this genus was emended.

### Taxonomy

#### Erection of New Genera

*Epigeocarpum* Błaszk., B.T. Goto, Jobim, Niezgoda & Marguno, gen. nov.

MycoBank No. MB838879.

*Etymology*: Latin, *Epigeocarpum*, *Epigeo* (=soil surface) and *carpum* (=fruit body), referring to the habitat, in which the type species of the new genus formed glomerocarps (=sporocarps).

*Type species*: *E. crypticum* Jobim, Błaszk., Niezgoda, Magurno & B.T. Goto

*Diagnosis*: Differs from *K. bistrata*, the phylogenetically closely related species of the monospecific genus *Kamienskia*, in (*i*) producing compact, epigeous, yellow-colored glomerocarps with a peridium and hyaline to light yellow, glomoid spores having a three-layered spore wall, of which the laminate layer 2 is relatively thick compared to the small spores size, contracts and, consequently, transfers into a crown-like structure in spores crushed in PVLG-based mountants and stains in Melzer’s reagent; (*ii*) the possession of a septum separating the spore subtending hyphal lumen from the spore interior; and (*iii*) the nucleotide composition of sequences of the 18S-ITS-28S nuc rDNA region and the *rpb1* gene.

*Genus description*: Producing glomoid glomerocarps in a compact epigeous unorganized glomerocarp. Spores hyaline to light yellow (4A4), usually globose to subglobose, 34–46 μm diameter, with a spore wall consisting of three permanent, smooth layers. Spore wall layer (swl) 2 laminate, usually transferring into a crown-like structure due to contracting in spores crushed in PVLG and PVLG + Melzer’s reagent. Only swl 2 stains in Melzer’s reagent. Subtending hypha funnel-shaped with a wall continuous with swl 1 and 2 and an open pore at the spore base; the channel connecting the lumen of the subtending hypha with the interior of spores closed by a septum continuous with swl 3; the septum usually positioned at half the thickness of swl 2; the subtending hyphal lumen gradually narrowing in maturing spores due to thickening of subtending hyphal wall layer 2.

*Silvaspora* Błaszk., Niezgoda, B.T. Goto, Crossay & Magurno, gen. nov.

MycoBank number MB838881.

*Type species*: *S. neocaledonica* (D. Redecker, Crossay & Cilia) Błaszk., Niezgoda, B.T. Goto, Crossay & Magurno, comb. nov. MycoBank MB838882.

*Specimens examined*: 3,768–3,772 (LPPDSE).

*Etymology*: *Silvaspora*, in honor of Dr. Gladstone Alves da Silva, Departamento de Micologia, CCB, Universidade Federal de Pernambuco, Brazil, in recognition of his important contribution to taxonomy and ecology of arbuscular mycorrhizal fungi.

*Diagnosis*: Differs from other genera in the Glomeraceae mainly in the nucleotide composition of sequences of the 18S-ITS-28S nuc rDNA region and the *rpb1* gene.

*Genus description*: Forming colored glomoid glomerospores with a spore wall consisting of three layers, of which only layer 1, forming the spore surface, is impermanent and hyaline to brightly colored. Subtending hypha colored similarly to the spore wall, cylindrical, slightly funnel-shaped, or constricted at the spore base, with a pore occluded due to thickening subtending hyphal wall layer 2 continuous with the laminate spore wall layer 2, rarely slightly open. Forming mycorrhiza with arbuscules, vesicles, and hyphae staining dark in trypan blue.

#### Emendation of the Genus *Dominikia* Błaszk., Chwat & Kovács Emend. Błaszk., Niezgoda, Magurno, Jobim & B.T. Goto

*Genus description*: Fungi forming glomoid-like spores in soil or epigeous glomerocarps (=sporocarps). Hypogeous spores usually produced in unorganized (with random distribution), loose to compact clusters, rarely singly, sometimes inside roots. Epigeous sporocarps compact with hundreds to thousands of randomly distributed glomerospores. Spores hyaline to yellow brown, small, 17–87 μm diameter when globose. Spore wall with two to three layers. Layer 1, forming the spore surface, mucilaginous, short-lived, or unit (not divided into sublayers), semipermanent to permanent, only slightly or not deteriorating with age. Layer 2 unit or laminate, permanent. Layer 3 flexible to semiflexible, permanent. Layers 1 and 3 staining or not staining in Melzer’s reagent. Spore subtending hypha cylindrical to funnel-shaped with a pore open or occluded by a septum (i) continuous with the innermost lamina(e) of the laminate spore wall layer 2 or 3, (ii) connecting the inner surfaces of the innermost subtending hyphal wall layer, (iii) continuous with the flexible to semiflexible innermost spore wall layer, and (iv) sometimes as in (iii) and still by another underlying septum connecting the inner surfaces of the innermost subtending hyphal wall layer. Forming vesicular–arbuscular or only arbuscular mycorrhiza staining dark in Trypan blue.

*Type species*: *Dominikia minuta* (Błaszk., Tadych & Madej) Błaszk., Chwat & Kovács Mycotaxon 76, 189. 2000.

*Other accepted species. Dominikia achra* (Błaszk., D. Redecker, Koegel, Schützek, Oehl & Kovács) Błaszk., Chwat & Kovács.

*Basionym*. *Glomus achrum* Błaszk., D. Redecker, Koegel, Schützek, Oehl & Kovács, Botany 87, 262. 2009.

*Dominikia aurea* (Oehl & Sieverd.) Blaszk. Chwat, G.A. Silva & Oehl.

*Basionym*. *Glomus aureum* Oehl & Sieverd. J. Appl. Bot., Angew. Bot. 77, 111. 2003.

*Dominikia bernensis* Oehl, Palenz., Sánchez-castro & G.A. Silva.

*Dominikia bonfanteae* Magurno, Niezgoda, B.T. Goto & Błaszk.

*Dominikia compressa* (Sieverd. Oehl, Palenz., Sánchez-Castro & G.A. Silva) Oehl, Palenz., Sánchez-Castro & G.A. Silva.

*Basionym. Glomus compressum* Sieverd., Oehl, Palenz., Sánchez-Castro & G.A. Silva, Nova Hedwigia 99, 433. 2014.

*Dominikia difficilevidera* Błaszk., Góralska & Chwat.

*Dominikia disticha* Błaszk., Chwat & Kovács.

*Dominikia duoreactiva* Błaszk., Góralska & Chwat.

*Dominikia indica* (Błaszk., Wubet & Harikumar) Błaszk., G.A. Silva & Oehl.

*Basionym*. *Glomus indicum* Błaszk., Wubet & Harikumar, Botany 88, 134. 2010.

*Dominikia iranica* (Błaszk., Kovács & Balázs) Błaszk., Chwat & Kovács.

*Basionym*. *Glomus iranicum* Błaszk., Kovács & Balázs, Mycologia 102, 1457. 2010.

*Dominikia lithuanica* Błaszk., Chwat & Góralska.

#### Description of New Species and a New Combination

**FIGURE 4:**
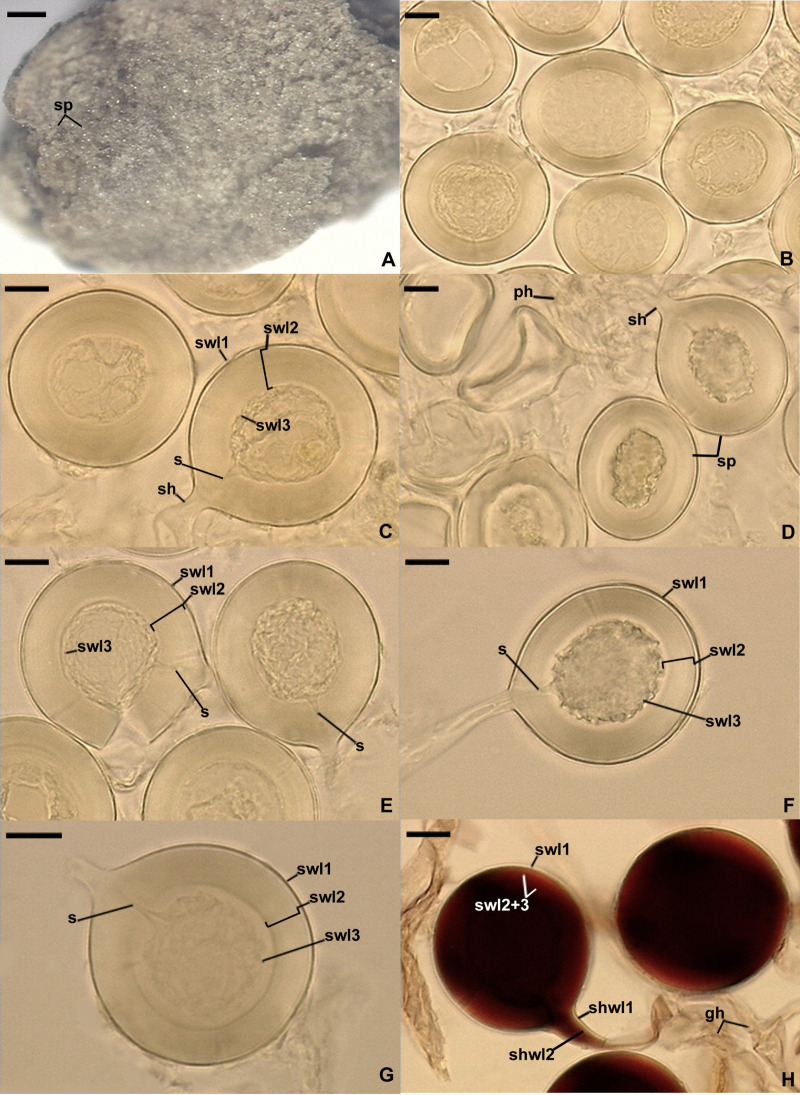
*Dominikia glomerocarpica*. **(A)** Glomerocarp (=sporocarp) with glomerospores (=spores; sp). **(B)** Intact spores. **(C–G)** Intact and crushed spores with a three-layered spore wall (swl1–3) and a subtending hypha (sh); note that swl2 did not change in shape in crushed spores, and swl3 usually slightly contracted and separated from the lower surface of the laminate swl2 in intact and crushed spores and formed a septum (s) located at approximately half the length of the channel connecting the subtending hyphal lumen with the spore interior; peridial hyphae (ph) are visible in **(D)**. **(H)** Intact spores with swl1–3, subtending hyphal wall layers (shwl) 1 and 2, and glebal hyphae (gh) mounted in PVLG + Melzer’s reagent. **(A)** Dry specimen. **(B–G)** Spores in PVLG. **(H)** Spores in PVLG + Melzer’s reagent. **(A)** Light microscopy. **(B–H)** Differential interference microscopy. Scale bars: **(A)** = 200 μm, **(B–H)** = 10 μm.

*Dominikia glomerocarpica* Jobim, Błaszk., Niezgoda, Magurno & B.T. Goto, sp. nov. Figures 4A–H.

MycoBank No. MB838883.

*Etymology*: Latin, *glomerocarpica*, referring to the glomerocarps (=sporocarps) produced by the species.

*Specimens examined*: Brazil, Parque das Trilhas, belonging to the Serra de Baturité Environmental Protection Area (4°16′36.8′′ S, 38°56′20.4′′ W; 865 m above sea level) in the Guarapiranga municipality, Ceará State, a glomerocarp found by K. Jobim in July 2018. Holotype. A fragment of a glomerocarp and a slide with spores no. UFRN-Fungos 3282, isotypes: a vial with a fragment of a glomerocarp no. 3802 and slides with spores no. 3773–3782 (LPPDSE).

*Diagnosis*: Differs from *D. aurea*, the sister phylogenetic relative, in (i) the habitat of production and the features of the structure forming the spore conglomeration; (ii) spore color, the composition of the spore wall and the phenotypic and biochemical properties of the spore wall layers; (iii) the characters of the septum separating the spore subtending hyphal lumen from the spore interior; and (iv) the nucleotide composition of sequences of the 18S-ITS-28S nuc rDNA region and the *rpb1* gene.

*Description*: Glomerospores formed in a compact epigeous glomerocarp. *Glomerocarp* yellowish gray (4B2) to grayish yellow (4B3); approximately 3.30 × 3.38 mm (Figure 4A). *Peridium* thin, hyaline to pale gray (1B1), only partially covering glomerospores’ conglomerations. *Gleba* yellowish white (4A2) to grayish yellow (4B3), with hyaline; straight or branched hyphae; (1.6−)3.2(−4.8) μm wide, with a wall 0.8–1.1 μm thick; staining pinkish white (7A2) to pastel red (9A6) in Melzer’s reagent; glomerocarp hosting hundreds of glomerospores (=spores; Figures 4B–H). *Spores* arise blastically at tips of sporogenous hyphae (Figures 4C,D,F–H). Spores hyaline to light yellow (4A5); globose to subglobose; (30−)39(−46) μm diameter; rarely ovoid; 34–39 μm × 40–46 μm; with one subtending hypha (Figures 4B–H). *Spore wall* composed of three permanent, smooth layers (Figures 4C,E–H). Layer 1, forming the spore surface, uniform (not containing visible sublayers), semiflexible, hyaline, (0.8−)1.2(−1.5) μm thick, tightly adherent to the upper surface of layer 2 (Figures 4C,E–H). Layer 2 laminate, semiflexible, hyaline to light yellow (4A5), (1.8−)8.2(−10.5) μm thick; consisting of very thin, <0.5 μm thick, laminae, tightly adherent to each other, not separating even in vigorously crushed spores; not contracting in spores crushed in PVLG-based mountants (Figures 4C,E–H). Layer 3 flexible, hyaline, approximately 1.0 μm thick, usually slightly contracting and separating from the lower surface of layer 2 in intact spores and spores crushed in PVLG, except for its funnel-shaped part associated with the inner surfaces of the laminate layer 2 forming the channel that connect the spore interior with the lumen of the subtending hypha (Figures 4C,E–H). In Melzer’s reagent, only spore wall layer 2 stains pastel red (9B7) to brownish violet (11D8; Figure 4H). *Subtending hypha* hyaline to light yellow (4A5); straight or recurved, funnel-shaped; (4.0−)5.8(−8.5) μm wide at the spore base (Figures 4C–H); not braking in crushed spores. *Wall of subtending hypha* hyaline to light yellow (4A5); (2.0−)2.5(−3.2) μm thick at the spore base; consisting of two layers continuous with spore wall layers 1 and 2 (Figure 4H). *Pore* (0.6−)0.9(−1.4) μm wide and open at the spore base; the channel connecting the lumen of the subtending hypha with the interior of spores closed by a septum continuous with spore wall layer (swl) 3; the septum usually positioned at half the thickness of swl 2; the subtending hyphal lumen gradually narrowing in maturing spores due to thickening of subtending hyphal wall layer 2 (Figures 4C,E–G). Spore content of hyaline oily substance. *Germination* unknown.

*Mycorrhizal associations*: no molecular analyses were performed on roots of the plant species that grew in the place where the glomerocarp of *D. glomerocarpica* was found. Attempts to grow *D. glomerocarpica* in single-species cultures with *P. lanceolata* as host plant failed.

*Distribution and habitat*: the Parque das Trilhas, being part of the Serra de Baturité Environmental Protection Area in Brazil, is so far the sole known site of occurrence of *D. glomerocarpica*. The geographic location and climate of this site were characterized in Section “Materials and Methods.”

BLAST searches indicated that *D. glomerocarpica* has not been found in other environments around the word before. The highest identities of 18S-ITS-28S and *rpb1* sequences of this species compared with sequences of the two loci deposited in GenBank were only 93.78 and 95.84%, respectively.

**FIGURE 5:**
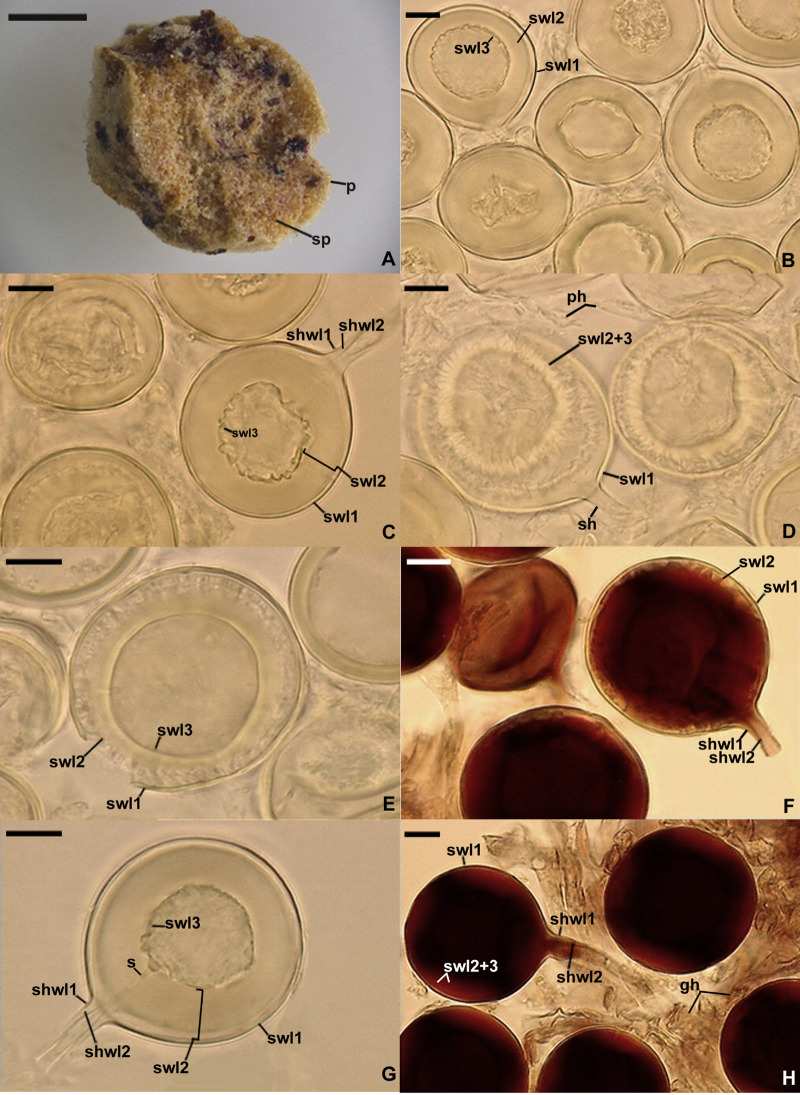
*Epigeocarpum crypticum*. **(A)** Glomerocarp (=sporocarp) with glomerospores (=spores; sp). **(B)** Spores on which no pressure was applied; note that spore wall layer (swl) 2 slightly contracted and separated from the lower surface of swl1, and swl3 only slightly separated from the lower surface of swl2. **(C–G)** Spore wall layers (swl) 1–3 and subtending hyphal wall layers (shwl) 1 and 2 of spores on which pressure was applied; note that the laminate swl2 transferred into a crown-like structure, and swl3 usually slightly contracted and separated from the lower surface of swl2 and formed a septum (s) located at approximately half the length of the channel connecting the subtending hyphal lumen with the spore interior; peridial hyphae (ph) are visible in **(D)**. **(H)** Intact spores with swl1–3, shwl1 and 2, and glebal hyphae (gh) mounted in PVLG + Melzer’s reagent. **(A)** Dry specimen. **(B–E,G)** Spores in PVLG. **(F,H)** Spores in PVLG + Melzer’s reagent. **(A)** Light microscopy. **(B–H)** Differential interference microscopy. Scale bars: **(A)** = 200 μm, **(B–H)** = 10 μm.

*Epigeocarpum crypticum* Jobim, Błaszk., Niezgoda, Magurno & B.T. Goto, sp. nov. Figures 5A–H.

MycoBank No. MB838880.

*Etymology*: Latin, *crypticum*, referring to the very small and hidden morphological differences compared to the very large differences in the DNA nucleoid composition between *E. crypticum* and its closest molecular relative, *K. bistrata*.

*Specimens examined*: Brazil, Parque das Trilhas, belonging to the Serra de Baturité Environmental Protection Area (4°16′36.8′′ S, 38°56′20.4′′ W; 865 m above sea level) in the Guarapiranga municipality, Ceará State, a glomerocarp found by K. Jobim in April 2018. Holotype: A fragment of a glomerocarp and a slide with spores no. UFRN-Fungos 3283, isotypes: a vial with a fragment of a glomerocarp no. 3803 and slides with spores no. 3783–3801 (LPPDSE).

*Diagnosis*: As that regarding the genus *Epigeocarpum* (see above).

*Description*: Glomerospores formed in a compact epigeous glomerocarp. *Glomerocarp* yellowish white (4A2) to grayish yellow (4B3); 1.65 mm × 1.69 mm (Figure 5A). *Peridium* thin, yellowish white (3A2) to pale yellow (3A3), only partially covering glomerospores’ conglomerations (Figure 5A). *Gleba* yellowish white (4A2) to light yellow (4A5), with hyaline; straight or branched hyphae; (2.2−)5.1(−7.5) μm wide, with a wall 0.6–1.8 μm thick; staining reddish white (7A2) to grayish red (8B6) in Melzer’s reagent; glomerocarp hosting hundreds of glomerospores (=spores; Figures 5A–H). *Spores* arise blastically at tips of sporogenous hyphae. Spores hyaline to light yellow (4A4); globose to subglobose; (34−)40(−46) μm diameter; rarely ovoid; 34–44 μm × 40–51 μm; with one subtending hypha (Figures 5B–H). *Spore wall* composed of three permanent, smooth layers (Figures 5B–H). Layer 1, forming the spore surface, uniform (not containing visible sublayers), semiflexible, hyaline, (0.8−)1.5(−2.0) μm thick, tightly adherent to layer 2 in spores immersed in water, but frequently slightly separated from the upper surface of layer 2, even in intact spores, due to contracting of layer 2 in PVLG-based mountants (Figures 5B–H). Layer 2 laminate, semiflexible, hyaline to light yellow (4A4), (2.0−)8.0(−12.5) μm thick; consisting of very thin, <0.5 μm thick, laminae, tightly adherent to each other, not separating even in vigorously crushed spores; usually transforming into a crown-like structure due to contracting in spores crushed in PVLG and PVLG + Melzer’s reagent (Figures 5C–H). Layer 3 flexible, hyaline, approximately 1.0 μm thick, usually slightly contracting and separating from the lower surface of layer 2 in intact spores and spores crushed in PVLG, except for its funnel-shaped part associated with the inner surfaces of the laminate layer 2 forming the channel that connect the spore interior with the lumen of the subtending hypha (Figures 5C–E,G,H). In Melzer’s reagent, only spore wall layer 2 stains reddish white (7A2) to brownish violet (11D8; Figures 5F,H). *Subtending hypha* hyaline to light yellow (4A4); straight or recurved, funnel-shaped; (4.4−)6.6(−9.0) μm wide at the spore base (Figures 5C,D,F–H); not braking in crushed spores. *Wall of subtending hypha* hyaline to light yellow (4A4); (2.0−)2.8(−3.3) μm thick at the spore base; consisting of two layers continuous with spore wall layers 1 and 2 (Figures 5C,F,G,H). *Pore* (0.6−)1.1(−1.6) μm wide and open at the spore base; the channel connecting the lumen of the subtending hypha with the interior of spores closed by a septum continuous with spore wall layer (swl) 3; the septum usually positioned at half the thickness of swl 2; the subtending hyphal lumen gradually narrowing in maturing spores due to thickening of subtending hyphal wall layer 2 (Figure 5G). Spore content of hyaline oily substance. *Germination* unknown.

*Mycorrhizal associations*: No molecular analyses were performed on roots of the plant species that grew in the place where the glomerocarp of *E. crypticum* was found. Attempts to grow *E. crypticum* in single-species cultures with *P. lanceolata* as host plant failed.

*Distribution and habitat*: The physical presence of *E. crypticum* was so far found only in Parque das Trilhas. The geographic location of the park and other characteristics of the habitat, in which the glomerocarp of *E. crypticum* was discovered, are described in *Distribution and habitat* regarding *D. glomerocarpica* (see above).

BLAST’s searches suggested that *E. crypticum* was also associated with roots of native plants growing in a petroleum-polluted soil of the Amazon region of Ecuador. The identity of the 28S MH504057 sequence compared to the 18S-ITS-28S sequences of *E. crypticum* was 98.5%. The identity of *E. crypticum rpb1* sequences compared to those listed by BLAST was only ≤92.7%.

**FIGURE 6:**
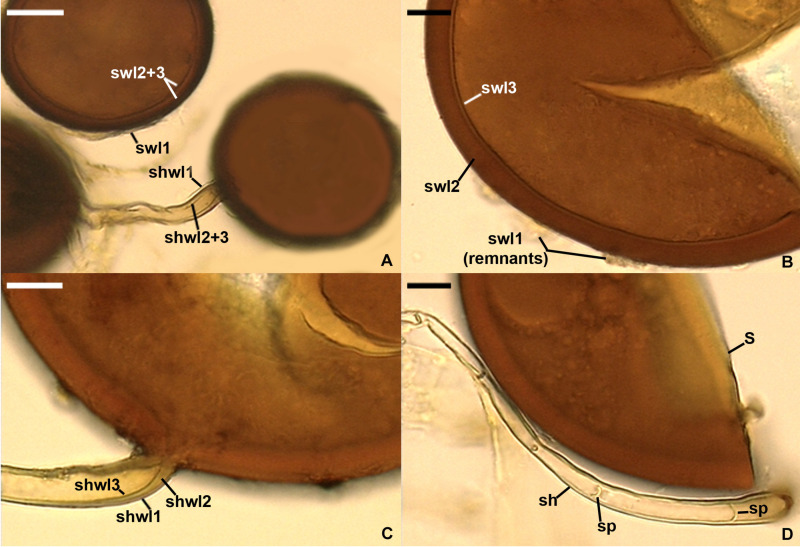
*Silvaspora neocaledonica*. **(A)** Spores with a spore wall (sw) consisting of three layers (swl1–3). **(B)** Spore wall layers (swl) 1–3; swl1 is almost completely sloughed off. **(C)** Subtending hyphal wall layers (shwl) 1–3. **(D)** Spore (s) and subtending hypha (sh) with two septa (sp) indicated. **(A,B,D)** Spores in PVLG. **(C)** Spores in PVLG + Melzer’s reagent. **(A–D)** Differential interference microscopy. Scale bars: **(A)** = 20 μm, **(B–D)** = 10 μm.

*Silvaspora neocaledonica* (D. Redecker, Crossay & Cilia) Błaszk., Niezgoda, B.T. Goto, Crossay & Magurno, comb. nov. Figures 6A–D.

MycoBank number MB838882.

*Basionym*: *Rhizophagus neocaledonicus* D. Redecker, Crossay & Cilia, Mycol. Prog. 17, 739. 2018.

*Synonym*: *Rhizophagus neocaledonicum* (D. Redecker, Crossay & Cilia) Oehl, Turrini & Giovann. Mycol. Prog. 17, 1218. 2018.

*Description*: Glomerospores formed singly and in loose clusters of 2–3 in soil, roots, and in root organ culture. *Spores* dark chestnut to coffee brown [orange (6C7) to brown (6E8)]; usually globose to subglobose; (61–)75(−90) μm diameter; sometimes oblong to irregular; 68–82 μm × 95–113 μm; with one subtending hypha (Figures 6A–C). *Spore wall* composed of three layers (Figures 6A,B). Layer 1, forming the spore surface, evanescent, short-lived, hyaline to yellowish white (4A2), 1.0–2.5 μm thick, usually highly deteriorated or completely sloughed off in mature spores. Layer 2 laminate, permanent, dark brown [orange (6C7) to brown (6E8)], (4.2–)6.1(−8.8) μm thick. Layer 3 uniform (without visible sublayers), bright brown [golden yellow (5B7) to orange (6B7)], 0.8–1.2 μm thick, usually tightly adherent to the lower surface of layer 2, even in vigorously crushed spores, difficult to detect and characterize due to the similarity in appearance to and almost inseparability from the innermost laminae of the laminate layer 2. None of spore wall layers 1–3 stains in Melzer’s reagent. *Subtending hypha* pale orange (5A3) to light orange (5A4); cylindrical, slightly funnel-shaped, or slightly constricted at the spore base; (6.2−)8.3(−11.6) μm wide at the spore base (Figures 6A,C,D). *Wall of subtending hypha* pale orange (5A3) to light orange (5A4); (1.6−)2.8(−5.4) μm thick at the spore base; consisting of three layers continuous with spore wall layers 1–3; subtending hyphal wall (shwl) layer 3 usually is difficult to see because it is thin, similarly colored, and tightly adherent to shwl2 (Figures 6A,C). *Pore* usually occluded due to thickening of spore wall layer 2 and subtending hyphal wall layer 2 (Figures 6C,D), rarely slightly open at the spore base; the subtending hyphal lumen occasionally with some transverse septa widely distributed along the subtending hypha (Figure 6D). *Germination* through subtending hypha. *Mycorrhiza* with arbuscules, vesicles, and hyphae staining lightly to dark in trypan blue.

*Notes*: The description presented above was prepared based on the original description of this species and examination of its spores provided by T. Crossay. We added color names of spores and spore wall layers determined from Kornerup and Wanscher (1983) to compare them with the color of the subtending hypha, whose pigmentation was not characterized in the original description of *S. neocaledonica*. Also, we changed some measurement values when they differed from the range of measurements given by Crossay et al. (2018) and replaced the name of spore wall layer 1, originally determined as mucilaginous, into evanescent. A mucilaginous spore wall layer *sensu* Stürmer and Morton (1997) stains in Melzer’s reagent, whereas spore wall layer 1 of *S. neocaledonica* does not react in this reagent. Finally, we significantly extended the characterization of the subtending hypha compared to that originally published.

*Specimens examined*: 3768–3772 (LPPDSE).

*Distribution and habitat*: To date, this species is known only from the type locality: Noumea, in the rhizosphere soil of *Alphitonia neocaledonica* (Schltr.) Guillaumin growing in an ultramafic *maquis* in the New Caledonia (Crossay et al., 2018). BLAST searches of 18S-ITS-28S and *rpb1* sequences indicated that *S. neocaledonica* has not been recorded in other regions of the world.

## Discussion

As we expected based on exploratory morphological and molecular phylogenetic analyses, our extended phylogenetic analyses of sequence alignments containing representatives of all genera of the Glomeraceae, using 18S-ITS-28S and *rpb1* data, unambiguously and strongly confirmed our hypothesis that the two glomerocarps found in Northeast Brazil are two new species of the Glomeromycota (Figures 1–3). Surprisingly, these morphologically almost identical fungi represent strongly phylogenetically divergent taxa in the Glomeraceae, one belonging to *Dominikia* and the second neighboring the monospecific genus *Kamienskia* (Figures 1–3). The divergences of 18S-ITS-28S and *rpb1* sequences between the new species, *D. glomerocarpica* and *E. crypticum*, were 18.5–19.0% and 11.0–11.8%, respectively.

Another interesting finding in our analyses was that the sister species of *D. glomerocarpica* was *D. aurea* (Figures 1–3), which presents significant morphological differences. *D. glomerocarpica* is distinguished morphologically by (i) the formation of a compact, epigeous glomerocarp with a peridium; (ii) the production of small, colorless to light yellow (4A4), glomoid spores; (iii) the possession of a spore wall consisting of three permanent, smooth layers, of which the colorless to light yellow (4A4) laminate layer 2 is distinctly thick compared to the spore size and stains dark in Melzer’s reagent; and (*iv*) the formation of an invaginated septum continuous with spore wall layer 3, which is usually located at half the length of the channel connecting the lumen of the spore subtending hypha with the spore interior (Figures 4A–H).

*D. aurea* was originally described to produce hypogeous, peridium-free, 450–1,200 μm × 510–1,600 μm diameter, glomerocarps (called sporocarps), consisting of tightly packed spores. However, the microphotographs presented in the article of Oehl et al. (2003) and our specimens testify that *D. aurea* spores arise mainly in compact, hypogeous clusters (Błaszkowski, 2012) rather than in typical unorganized glomerocarps, as the much larger, epigeous glomerocarp with a peridium formed by *D. glomerocarpica*. Also, we often extracted loose clusters with few spores and single spores of *D. aurea*. Moreover, *D. aurea* spores are much darker colored and never are colorless at maturity, have a 1.2- to 2.4-fold thinner and only two-layered spore wall, of which layer 1 is impermanent, usually completely sloughed off in mature spores, and stains in Melzer’s reagent. Finally, the pore of the spore subtending hypha is closed by a curved septum, which is continuous with the innermost laminae of the laminate spore wall layer 2 and is located at or up to 5 μm below the spore base (Oehl et al., 2003; Błaszkowski, 2012). The clear distinctiveness of *D. aurea* and *D. glomerocarpica* also proved the large molecular divergences between their 18S-ITS-28S and *rpb1* sequences, which were 8.5–10.2% and 4.3–4.8%, respectively.

Another surprising finding of our efforts to establish the taxonomic status of the Brazilian fungi was the placement of the previously named “Species 2” in a sister clade to the monospecific, generic clade represented by *K. bistrata* (Figures 1–3). However, the two species differ fundamentally in morphology and are separated from one another by a very large molecular distance (11.0–15.5%). Therefore, we erected a new genus for “Species 2,” named *Epigeocarpum*, typified by *E. crypticum*, despite the fact that the morphological characters distinguishing and clearly separating this species from *K. bistrata* are shared by other members of the Glomeraceae, as discussed below.

The distinctive morphological features of *E. crypticum* are (i) the production of small, colorless or light yellow (4A5), glomoid spores in a compact, epigeous glomerocarp with a peridium; (ii) a spore wall composed of three permanent, smooth layers, of which the much thicker laminate layer 2 stains dark in Melzer’s reagent and, most importantly, contracts and transfers into a crown-like structure in spores crushed in PVLG and PVLG + Melzer’s reagent; and (iii) the formation of a septum by spore wall layer 3 of characters and location similar to those of the septum of *D. glomerocarpica* spores (Figures 5A–H).

*K. bistrata* does not produce epigeous glomerocarps with a peridium, but its spores are grouped in loose to compact hypogeous clusters (Błaszkowski et al., 2009a; Błaszkowski, 2012). Most importantly, the spore wall of *K. bistrata* is 2.2- to 4.4-fold thinner than the *E. crypticum*’*s*, consists of only two colorless layers, of which the uniform layer 1 is thicker than the laminate layer 2, none of these layers stains in Melzer’s reagent, and the channel connecting the lumen of the spore subtending hypha with the spore interior is not closed by any septum.

As mentioned above, one of the distinctive features of the two here newly described species is the location of the septum separating the lumen of the spore subtending hypha from the spore interior (Figures 4C,F,G, 5G). The septum usually occurs at half the thickness of the laminate spore wall layer 2 as similarly described for *Rhizoglomus maiae* (Błaszkowski et al., 2019b), whereas for all other glomoid spore-producing species, the septum is formed at or slightly below the spore base. Moreover, *R. maiae* also produces compact, epigeous clusters with spores, whose spore wall structure and phenotypic properties of its components are like those of *D. glomerocarpica* and *E. crypticum*, except for the contractility of spore wall layer 2 of *E. crypticum* (Figures 5D–F). Thus, the location of the septum in these three species, belonging to three far related genera of the Glomeraceae, clearly proves that this feature is phylogenetically uninformative.

The formation of the septum much above the spore base in *D. glomerocarpica*, *E. crypticum*, and *R. maiae* probably is associated with the spore wall ontogeny and particularly with the differentiation of the distinctively thick laminate spore wall layer 2 by these fungi. As ontogenetic studies of spores of *Claroideoglomus claroideum* revealed, the innermost spore wall layer 4, forming an invaginated septum at or slightly below the spore base, arises after the full differentiation of the laminate spore wall layer 3 (Stürmer and Morton, 1997). Most likely, a similar sequence of events occurs during the development of the spore wall of *D. glomerocarpica*, *E. crypticum*, and *R. maiae*. However, the laminate spore wall layer 3 of *C. claroideum* is a relatively thin component of the entire spore wall, and consequently, the full differentiation of this layer is faster than that of the much thicker laminate spore wall layer 2 of the three main species discussed above. Therefore, the formation of the invagination of spore wall layer 4 of *C. claroideum*, shaped by the available space (the channel connecting the spore subtending hyphal lumen with the spore interior) in which the development of this layer is initiated, starts relatively earlier and closer to the spore base.

Another structure distinguishing *E. crypticum* is the contracting laminate spore wall layer 2, which takes the shape of a crown in spores crushed in PVLG-based mountants (Figures 5D–F). A spore wall layer of similar properties was also found in *Microkamienskia perpusilla*, originally described as *Glomus perpusillum* (Błaszkowski et al., 2009b), although the contracting spore wall layer 2 of *M. perpusilla* is a unit structure (not divided into visible sublayers) and does not transform into a crown following crushing of spores in PVLG-based mountants. Therefore, it seems that the contractility of spore wall layer 2 of *E. crypticum* is also not a generic synapomorphy of the new genus *Epigeocarpum*.

In summary, the sole characters convincingly rendering the genus *Epigeocarpum* unique are those residing in its DNA. Importantly, the genetic differences between the 18S-ITS-28S (14.5–15.6%) and *rpb1* (11.0–11.6%) sequences of *E. crypticum* and *K. bistrata* are similar to those between sequences of directly neighboring species of most of the genera present in the tree depicted in Figures 1, 2. Several examples of pairwise comparisons are displayed in Supplementary Table 3. Of the described species producing unorganized glomerocarps with glomoid spores, whose molecular phylogeny is uncertain or unknown, *D. glomerocarpica* and *E. crypticum* most closely resemble *Glomus microcarpum* and *Glomus nanolumen*; the spores of these species are similar in color and size. The main differences readily separating the four species reside in their spore wall structure, the phenotypic and histochemical features of the spore wall components, and the characters of the pore connecting the subtending hyphal lumen with the spore interior.

*Glomus microcarpum* was originally characterized as having one, thick spore wall, which was also found by Gerdemann and Trappe (1974) and Berch and Fortin (1984). Instead, Błaszkowski (2012) defined and illustrated this species as producing spores with a spore wall consisting of two layers: a short-lived, quickly sloughing off, or semipermanent, slowly decomposing, thin layer 1, forming the spore surface, and a permanent, thick, laminate layer 2, staining dark red in Melzer’s reagent. Similar specimens identified as *G. microcarpum* with a spore wall as that characterized by Błaszkowski (2012) were presented by Oehl et al. (2011) and repeatedly found in Northeast Brazil (unpublished data). Of the Glomeromycota species grown in culture, only *Sieverdingia tortuosa* produced spores with a one-layered spore wall (Błaszkowski et al., 2019a), and in most of glomoid and glomoid-like spore-producing species, their laminate spore wall layer is covered with a relatively thin, usually short-lived, rarely permanent layer (Oehl et al., 2011; Błaszkowski, 2012). In field-collected spores, this outer spore wall layer is usually strongly or completely sloughed off, and therefore, it is difficult to detect or no longer present. On top of our knowledge, *G. microcarpum* has not yet been grown in culture, which makes impossible to examine spores at different developmental stages. However, regardless of the number of layers existing in the spore wall of the true *G. microcarpum*, the structure of the spore wall and the phenotypic properties of its components in *D. glomerocarpica* and *E. crypticum* (Figures 4B–H, 5B–H) differ clearly from those characterized above for *G. microcarpum*. The sequence identity, obtained by BLAST, of 18S-ITS-28S sequences of *E. crypticum* compared to 38 18S-ITS-28S sequences of *G. microcarpum* deposited in GenBank is only 87.5–92.02%. Unfortunately, no data were published about the morphology of the “donor” of the sequences ascribed to *G. microcarpum*. Thus, there is an urgent need for unambiguous morphological and molecular characterization of *G. microcarpum*, one of the first two described species currently classified in the phylum Glomeromycota and until 2010 regarded as the type species of *Glomus* (e.g., Gerdemann and Trappe, 1974).

Regarding *G. nanolumen*, its glomerocarps do not contain a peridium, and most importantly, their spores have a two-layered spore wall only, of which the laminate layer 2 is not uniform in thickness but differs two to three times in its different regions, and none of these layers stains in Melzer’s reagent (Koske and Gemma, 1989; Błaszkowski, 2012). Moreover, the subtending hypha of *G. nanolumen* is 1.6- to 2.5-fold wider at the spore base, and the channel connecting the subtending hyphal lumen with the spore interior is open.

Three evidences unambiguously justified the erection of the new monospecific genus *Silvaspora* in the Glomeraceae for the New Caledonian fungus, originally described as *R. neocaledonicus* (Crossay et al., 2018) and here renamed *S. neocaledonica*. First, our phylogenetic analyses of the 18S-ITS-28S and 18S-ITS-28S + *rpb1* alignments, containing sequences of all sequenced *Rhizoglomus* species, placed the New Caledonian fungus in a clade at the rank of genus, and importantly, the clade was located in the sister relation to the genus *Sclerocystis*, which was outside the *Rhizoglomus* clade (Figures 1, 3). Thus, assigning the New Caledonian fungus to *Rhizoglomus* would make this genus polyphyletic. Moreover, although the analyses of *rpb1* sequences clustered the New Caledonian fungus with *Rhizoglomus* spp., this grouping did not obtain either BI or ML support (Figure 2). Second, the divergences of 18S-ITS-28S and *rpb1* sequences of the New Caledonian fungus from those of *S. sinuosa* were 22.7–22.9% and 11.4%, respectively. Thus, these divergence values were similar to the sequence divergences of other neighboring genera in the Glomeraceae (see above). Third, the mode of formation and morphology of spores of *S. sinuosa* differ fundamentally from those of *S*. *neocaledonica* (Gerdemann and Bakshi, 1976; Błaszkowski, 2012; Crossay et al., 2018; pers. observ.). All *Sclerocystis* species *sensu* Schüßler and Walker (2010) produce organized glomerocarps, in which glomoid-like spores arise radially from a central sterile plexus of mycelium, and the spores have a one-layered spore wall. Instead, *S*. *neocaledonica* produces spores singly or in clusters of 2 to 3, and their spore wall consists of three layers (Figures 6A–D; Crossay et al., 2018; pers. observ.).

We did not include into our phylogenetic analyses the monospecific genus *Simiglomus* with *Simiglomus hoi* because it was erected based on two sequences of the 18S gene (Oehl et al., 2011), which in our alignments is represented only by approximately 240 base pairs, and the origination of these sequences is uncertain, as stated by Redecker et al. (2013). Thus, further studies are needed to clarify the status of *Simiglomus* in the Glomeraceae.

The known glomerocarpic species probably constitute a small part of those existing in nature, mainly due to the difficulty in finding and characterizing these fungi. Collecting glomerocarps by raking and searching the upper soil layer and buried plant fragments is much more time-consuming and tiring than the wet-sieving and decanting method (Gerdemann and Nicolson, 1963) commonly used to find specimens of the Glomeromycota. Interestingly, Gerdemann and Trappe (1974) stated that finding glomerocarps is also related to the acquisition of a so-called hypogeous instinct. Attempts to grow glomerocarpic fungi in culture rarely succeeded, and therefore their characterization, defined only on the basis of field-collected specimens, may lack accurateness. Besides, the morphological and molecular characterization of these fungi may be impossible due to the degradation of their physical structures and DNA (pers. observ.). Importantly, the few described glomerocarpic species with known molecular phylogeny belong to various taxonomic groups in the Glomeromycota, and frequently they miss genus-specific morphological characters. Moreover, molecular phylogenetic analyses have shown that glomerocarpic-like fruit bodies with glomoid-like spores are also produced by fungal taxa not belonging to the Glomeromycota (Yamamoto et al., 2020). Also, the trophic status of glomerocarpic and glomerocarpic-like species is unclear, particularly that of epigeocarpic species. In order to know the real representativeness of glomerocarpic species in the Glomeromycota and determine their trophic and taxonomic status, the collection of specimens of this group of fungi and the study of their ecology and phylogeny should be significantly intensified.

## Data Availability Statement

The datasets generated for this study can be found in online repositories. The names of the repository/repositories and accession number(s) are as follows: GenBank MW507148-MW507157, MW541060-MW541067, and MW249072-MW249073.

## Author Contributions

BG, FM, JB, PN, and SZ were responsible for conceptualization and funding acquisition. BG, FM, JB, and PN were responsible for data acquisition. FM, JB, PN, TB, and WB performed analyses. JB and FM wrote the first draft. BG, EM, FM, JB, KJ, LC, PN, RM, PM, SZ, TC, and WB wrote, reviewed, and edited the manuscript. All authors have read and agreed to the published version of the manuscript.

## Conflict of Interest

The authors declare that the research was conducted in the absence of any commercial or financial relationships that could be construed as a potential conflict of interest.

## Footnotes

1 https://eu.idtdna.com

2 http://maft.cbre.jp/alignment/server/

3 https://sites.google.com/site/cmzmasek/home/software/archaeopteryx-js
