# Supplementary material for: New Glomeromycotan Taxa, Dominikia glomerocarpica sp. nov. and Epigeocarpum crypticum gen. nov. et sp. nov. From Brazil, and Silvaspora gen. nov. From New Caledonia
Source: Front Microbiol. 2021 Apr 23;12:655910. doi: 10.3389/fmicb.2021.655910 (PMC8102679; doi:10.3389/fmicb.2021.655910)
Supplement: Supplementary file 1 [file Table_1.DOCX]

**Supplementary Table 1**. Names of fungal genera with abbreviations, species and their authors used in the paper.

| *Dominikia* (= *D.*) | Błaszk. et al. |
| --- | --- |
| *D. aurea* | (Oehl & Sieverd.) Błaszk. et al. |
| *D. bernensis* | Oehl et al. |
| *D. compressa* | (Sieverd. et al.) Oehl et al. |
| *D. difficilevidera* | Błaszk., Góralska & Chwat |
| *D. disticha* | Błaszk. et al. |
| *D. duoreactiva* | Błaszk., Góralska & Chwat |
| *D. emiratia* | Błaszk. et al. |
| *D. lithuanica* | Błaszk., Chwat & Góralska |
| *D. litorea* | Błaszk. & Kozłowska |
| *D. minuta* | (Błaszk. et al.) Błaszk. et al. |
| *Diversispora epigaea* (= *Di*.) | (B.A. Daniels & Trappe) C. Walker & A. Schüßler |
| *Di*. *sporocarpia* | Chachuła et al. |
| Entrophosporaceae | Oehl & Sieverd |
| *Funneliformis mosseae* | (T.H. Nicolson & Gerd.) C. Walker & A. Schüßler |
| Glomeraceae | Piroz. & Dalpé |
| *Glomus* (= *G*.) | Tul. & C. Tul. |
| *G. macrocarpum* | Tul. & C. Tul. |
| *G. arborense* | McGee |
| *G. microcarpum* | Tul. & C. Tul. |
| *G. nanolumen* | Koske & Gemma |
| *G. pallidum* | I.R. Hall |
| *G. tenerum* | P.A. Tandy |
| *G. warcupii* | McGee |
| *Halonatospora* (= *H*.) | Błaszk. et al. |
| *H.* *pansihalos* | (S.M. Berch & Koske) Błaszk. et al. |
| *Kamienskia* (= *K*.) | Błaszk. et al. |
| *K. bistrata* | (Błaszk. et al.) Błaszk. et al. |
| *K. divaricata* | Błaszk., Chwat & Góralska |
| *K. perpusilla* | (Błaszk. & Kovács) Błaszk., Chwat & Kovács |
| *Microkamienskia* (= *M*.) | Corazon-Guivin, G.A. Silva & Oehl |
| *M. perpusilla* | (Błaszk. & Kovács) Corazon-Guivin, G.A. Silva & Oehl |
| *M. peruviana* | Corazon-Guivin, G.A. Silva & Oehl |
| *Microdominikia* | Oehl, Corazon-Guivin & G.A. Silva |
| *Nanoglomus* (= *N*.) | Corazon-Guivin, G.A. Silva & Oehl |
| *N. plukenetiae* | Corazon-Guivin, G.A. Silva & Oehl |
| *Orientoglomus* | G.A. Silva, Oehl & Corazon-Guivin |
| *Redeckera* (= *Re*.) *megalocarpa* | (D. Redecker) C. Walker & A. Schüßler |
| *Re. fulva* | (Berk. & Broome) C. Walker & A. Schüßler |
| *Re. pulvinata* | (Henn.) C. Walker & A. Schüßler |
| *Rhizoglomus* (= *R*.) *irregulare* | (Błaszk. et al.) Sieverd., G.A. Silva & Oehl |
| *R. clarum* | (T.H. Nicolson & N.C. Schenck) Sieverd., G.A. Silva & Oehl |
| *R. dalpeae* | Błaszk. et al. |
| *R. dunense* | Błaszk. & Kozłowska |
| *R. maiae* | Jobim et al. |
| *R. neocaledonicum* | (D. Redecker, Crossay & Cilia) Oehl, Turrini & Giovann. |
| *Rhizophagus* | P.A. Dang. |
| *Rhizophagus neocaledonicus* | D. Redecker, Crossay & Cilia |
| *Sclerocarpum amazonicum* | Jobim et al. |
| *Sclerocystis* | Berk. & Broome |
| *Sclerocystis sinuosa* | Gerd. & B.K. Bakshi |
| *Septoglomus constrictum* | (Trappe) Sieverd., G.A. Silva & Oehl |
| *Sieverdingia tortuosa* | (N.C. Schenck & G.S. Sm.) Błaszk., Niezgoda & B.T. Goto |
| *Simiglomus* | Sieverd., G.A. Silva & Oehl |
| *Similglomus hoi* | (S.M. Berch & Trappe) G.A. Silva, Oehl & Sieverd. |
