# Supplementary material for: New Glomeromycotan Taxa, Dominikia glomerocarpica sp. nov. and Epigeocarpum crypticum gen. nov. et sp. nov. From Brazil, and Silvaspora gen. nov. From New Caledonia
Source: Front Microbiol. 2021 Apr 23;12:655910. doi: 10.3389/fmicb.2021.655910 (PMC8102679; doi:10.3389/fmicb.2021.655910)
Supplement: Supplementary file 2 [file Table_2.DOCX]

| **Supplementary Table 2**. Pairwise comparison of sequences from neighbouring species | | |
| --- | --- | --- |
|  |  | % of dissimilarity |
| **18S-ITS-28S locus** |  |  |
| *Funneliformis mosseae* FN547474 | *Septoglomus turnauae* KF060325 | 15.5 |
| *Funneliglomus sanmartinensis* MK348927 | *Septoglomus turnauae* KF060325 | 15.6 |
| *Microdominikia litorea* MG710520 | *Orientoglomus emiratium* KY555051 | 15.2 |
| *Microdominikia litorea* MG710519 | *Dominikia compressa* HG798899 | 19.4 |
| *Microkamienskia divaricata* KX758121 | *Kamienskia bistrata* KJ564135 | 20.1 |
| *Sclerocystis sinuosa* AJ133706-AJ437106-FJ461846 | *Rhizoglomus melanum* HG964397 | 25.4 |
|  |  |  |
| **rpb1 locus** |  |  |
| *Microdominikia litorea* MG710513 | *Orientoglomus emiratium* KY555044 | 11.1 |
| *Glomus tetrastratosum* MG710516 | *Microkamienskia divaricata* KX784773 | 12.9 |
| *Sclerocarpum amazonicum* MK036773 | *Glomus macrocarpum* KX784773 | 12.9 |
