# Supplementary material for: New Glomeromycotan Taxa, Dominikia glomerocarpica sp. nov. and Epigeocarpum crypticum gen. nov. et sp. nov. From Brazil, and Silvaspora gen. nov. From New Caledonia
Source: Front Microbiol. 2021 Apr 23;12:655910. doi: 10.3389/fmicb.2021.655910 (PMC8102679; doi:10.3389/fmicb.2021.655910)
Supplement: Supplementary file 3 [file Table_3.DOCX]

**Supplementary Table 3**. Accession numbers of rDNA and *rpb1* sequences used in the phylogenetic analysis. Accession numbers of concatenated sequences are shown in the same line. When available, the information about the isolate (i), strain (s) or voucher specimen (sv) is provided beside the accession number.

| Species | Accession number rDNA sequence | Isolate/strain/voucher-specimen | Accession number *rpb1* sequence | Isolate/strain/voucher-specimen |
| --- | --- | --- | --- | --- |
| *Claroideoglomus*  *claroideum* | FR750058 | "Att1063-4" (i) | HG316018 | "BEG23" (s) |
|  | FR750059 |  |  |  |
| *Claroideoglomus*  *drummondii* | AJ972464 | "Holotype.Blaszkowski,J.2578(DPP)" (sv) | MT733209 | “USK_D2" (i) |
|  | AJ972465 |  | MT733210 |  |
| *Claroideoglomus*  *walkeri* | KP191489 | "G162" (i) | MT733207 | "USK_C8" (i) |
|  | KP191490 |  | MT733208 |  |
| *Dominikia achra* | KJ564159 | "G146" (i) | KX784805 | "G146" (i) |
|  | KJ564157 |  | KX784807 |  |
|  | KJ564158 |  | KX784806 |  |
| *Dominikia aurea* | KM056657 | "Da" (i) | KX784792 | "Da" (i) |
|  | KM056659 |  | KX784793 |  |
|  | KM056658 |  |  |  |
|  | KM056661 |  |  |  |
| *Dominikia bernensis* | HG938302 | "FO310" (i) |  |  |
|  | HG938303 |  |  |  |
|  | HG938304 |  |  |  |
| *Dominikia*  *bonfanteae* | MW232906 | "USK_D5" (i) | MW249067 | "USK_D5" (i) |
|  | MW232910 |  | MW249068 |  |
|  | MW232905 |  |  |  |
|  | MW232909 |  |  |  |
| *Dominikia*  *compressa* | HG798895 | "FO352" (i) |  |  |
|  | HG798896 |  |  |  |
|  | HG79889 |  |  |  |
|  | HG798899 |  |  |  |
| *Dominikia*  *difficilevidera* | KR105644 | "G279" (i) | KX784795 | "G279" (i) |
|  | KR105645 |  | KX784796 |  |
|  | KR105646 |  | KX784797 |  |
| *Dominikia disticha* | KJ564147 | "G237" (i) | KX784786 | "G237" (i) |
|  | KJ564146 |  | KX784784 |  |
|  | KJ564148 |  | KX784787 |  |
| *Dominikia*  *duoreactiva* | KR105638 | "G271" (i) | KX784803 | "G271" (i) |
|  | KR105640 |  | KX784800 |  |
|  | KR105639 |  | KX784801 |  |
| *Dominikia*  *glomerocarpica* | MW507148 | field-collected sporocarps | MW541064 | field-collected sporocarps |
|  | MW507152 |  | MW541065 |  |
|  | MW507149 |  |  |  |
|  | MW507150 |  |  |  |
|  | MW507151 |  |  |  |
| *Dominikia indica* | KJ564166 | "G211" (i) | KX784799 | "G211" (i) |
|  | KJ564169 |  | KX784798 |  |
|  | KJ564167 |  |  |  |
|  | KJ564168 |  |  |  |
| *Dominikia iranica* | KJ564153 | "G187" (i) | KX784788 | "G187" (i) |
|  | KJ564154 |  | KX784790 |  |
|  | KJ564155 |  | KX784791 |  |
|  | KJ564156 |  | KX784789 |  |
| *Dominikia lithuanica* | KX758115 | "G331" (i) | KX784769 | "G331" (i) |
|  | KX758119 |  | KX784771 |  |
|  | KX758117 |  | KX784770 |  |
|  | KX758116 |  |  |  |
| *Dominikia minuta* | KJ564163 | "G137" (i) |  |  |
|  | KJ564164 |  |  |  |
|  | KJ564165 |  |  |  |
| *Entrophospora*  *infrequens* | MT722034 | “E.infrequens” (i) | MT733201 | “E.infrequens” (i) |
|  | MT722039 |  | MT733202 |  |
| *Epigeocarpum*  *crypticum* | MW507153 | field-collected sporocarps | MW541066 | field-collected sporocarps |
|  | MW507157 |  | MW541067 |  |
|  | MW507154 |  |  |  |
|  | MW507155 |  |  |  |
|  | MW507156 |  |  |  |
| *Funneliformis*  *mosseae* | FN547474 | "BEG12" (s) | JN985046 | "LPA34" (s) |
|  | FN547476 |  | JN985051 | "LPA34" (s) |
|  | FN547486 |  | JN985047 | "LPA58" (s) |
| *Funneliglomus*  *sanmartinensis* | MK348926 | "1a" (i) |  |  |
|  | MK348927 | "1b" (i) |  |  |
|  | MK348928 | "2a" (i) |  |  |
| *Glomus*  *macrocarpum* | FR750530 | "W5288" (s) | HG316006 | "Att1495-16" (i) |
|  | FR750526 | "W5288" (s) | HG316007 | "Att1495-16" (i) |
|  | FR750532 | "W5293" (s) | HG316021 | "Att1519-0" (i) |
| *Glomus*  *tetrastratosum* | KM056651 | “G276” (i) | MG710514 | “G276” (i) |
|  | KM056654 |  | MG710516 |  |
|  | KM056655 |  | MG710515 |  |
| *Halonatospora*  *pansihalos* | MH560600 | “G. pansihalos” (i) | MH560590 | “G. pansihalos” (i) |
|  | MH560604 |  | MH560591 |  |
|  | MH560601 |  | MH560592 |  |
| *Kamienskia bistrata* | KJ564135 | "G205" (i) | KX784777 | "G205" (i) |
|  | KJ564137 |  | KX784779 |  |
|  | KJ564133 |  | KX784778 |  |
|  | KJ564134 |  |  |  |
|  | KJ564136 |  |  |  |
| *Microdominikia*  *litorea* | MG710518 | "Dl-6" (i) | MG710511 | "G351" (i) |
|  | MG710520 | "Dl-B1" (i) | MG710512 |  |
|  | MG710517 | "Dl-10" (i) | MG710513 |  |
|  | MG710519 | "Dl-8" (i) |  |  |
| *Microkamienskia*  *divaricata* | KX758121 | "G240" (i) | KX784773 | "G240" (i) |
|  | KX758126 |  | KX784774 |  |
|  | KX758123 |  | KX784775 |  |
| *Microkamienskia*  *perpusilla* | KJ564139 | "G169" (i) | KX784781 | "G169" (i) |
|  | KJ564141 |  | KX784782 |  |
|  | KJ564143 |  | KX784783 |  |
|  | KJ564144 |  |  |  |
| *Microkamienskia*  *peruviana* | MK903006 |  |  |  |
|  | MK903007 |  |  |  |
|  | MK903008 |  |  |  |
| *Nanoglomus*  *plukenetiae* | MK875632 |  |  |  |
|  | MK875634 |  |  |  |
|  | MK875637 |  |  |  |
| *Oehlia diaphana* | MG836667 | DAOM227022 (s) | MG836656 | DAOM227022 (s) |
|  | MG836662 | DAOM745424 (s) | MG836657 | DAOM227022 (s) |
|  | MG836664 | DAOM227022 (s) | MG836654 | DAOM745424 (s) |
| *Orientoglomus*  *emiratium* | KY555052 | "G344" (i) | KY555043 | "G344" (i) |
|  | KY555053 |  | KY555044 |  |
|  | KY555051 |  | KY555045 |  |
| *Rhizoglomus*  *arabicum* | KF154765 | "F84" (i) |  |  |
|  | KF154766 |  |  |  |
|  | KF154767 |  |  |  |
| *Rhizoglomus*  *clarum* | FM865536 | "Att894-7" (i) | HG316004 | "WV235" (s) |
|  | FM865544 |  | HG316024 | "BEG142" (s) |
| *Rhizoglomus*  *custos* | GQ205076 | "DAOM 236361" (s) |  |  |
| *Rhizoglomus*  *dalpeae* | MN130951 | “G379” (i) | MW249072 | “G379” (i) |
|  | MN130953 |  | MW249073 |  |
| *Rhizoglomus*  *dunense* | KY555054 | "G232" (i) | KY555047 | "G232" (i) |
|  | KY555055 |  | KY555048 |  |
| *Rhizoglomus*  *fasciculatum* | FR750071 | "MUCL46100" (s) |  |  |
|  | FR750072 |  |  |  |
| *Rhizoglomus*  *intraradices* | FM865562 | "Att4-38" (i) | HG316020 | "MUCL49413" (s) |
|  | FM865559 | "Att4-38" (i) |  |  |
|  | FM865577 | "Att4-41" (i) |  |  |
| *Rhizoglomus*  *invermaium* | HG969378 | "Att1646" (i) |  |  |
|  | HG969381 |  |  |  |
| *Rhizoglomus*  *irregulare* | FM865550 | "Att1192-44" (i) | HG315983 | "LPA71-DAOM197198" (s) |
|  | FM865555 |  | HG315984 |  |
| *Rhizoglomus*  *maiae* | MN130955 | field-collected sporocarps |  |  |
|  | MN130956 |  |  |  |
| *Rhizoglomus*  *melanum* | HG964396 | "Dovre 6K" (i) |  |  |
|  | HG964397 |  |  |  |
|  | HG964398 |  |  |  |
| *Rhizoglomus*  *natalensis* | KJ210824 | "G261" (i) |  |  |
|  | KJ210826 |  |  |  |
| *Rhizoglomus*  *proliferum* | FM992401 | "MUCL41827" (s) | AM284981 | "DAOM226389" (s) |
|  | FM992398 |  | AM284982 |  |
| *Rhizoglomus*  *silesianum* | MN130961 | "USK_A5" (i) | MW541062 | "USK_A5" (i) |
|  | MN130958 |  | MW541063 |  |
| *Rhizoglomus*  *variabile* | MN384871 | "Rv2" (i) |  |  |
|  | MN384874 | "Rv5" (i) |  |  |
|  | MN384876 | "Rv7" (i) |  |  |
| *Rhizoglomus*  *venetianum* | LS974597 | "AT-2018" (i) |  |  |
|  | LS974598 |  |  |  |
| *Rhizoglomus*  *vesiculiferum* | MG836659 | "Rv” (i) |  |  |
|  | MG836660 |  |  |  |
| *Sclerocarpum*  *amazonicum* | MK036784  MK036781  MK036782  MK036783 | field-collected sporocarps | MK036773 | field-collected sporocarps |
|  |  |  |  |  |
|  |  |  |  |  |
|  |  |  |  |  |
| *Sclerocystis*  *sinuosa* | AJ133706+  AJ437106+  FJ461846 | "MD126" (i) | HG315990 | "MD126" (i) |
| *Septoglomus*  *turnauae* | KF060324 | "G243" (i) | MH560597 | "G243" (i) |
|  | KF060327 |  | MH560598 |  |
|  | KF060325 |  | MH560599 |  |
| *Silvaspora*  *neocaledonica* | KY362437 | "rh" (i) | MW541060 | "rh" (i) |
|  | KY362436 |  | MW541061 |  |
|  | KY362438 |  |  |  |
